# Influence of Different Intensities of Tillage on Physiological Characteristics and Productivity of Crop-Rotation Plants

**DOI:** 10.3390/plants11223107

**Published:** 2022-11-15

**Authors:** Daiva Janusauskaite, Grazina Kadziene

**Affiliations:** 1 Department of Plant Nutrition and Agroecology, Institute of Agriculture, Lithuanian Research Centre for Agriculture and Forestry, Instituto 1, LT-58344 Kedainiai, Lithuania; 2 Department of Soil and Crop Management, Institute of Agriculture, Lithuanian Research Centre for Agriculture and Forestry, Instituto 1, LT-58344 Kedainiai, Lithuania

**Keywords:** crops, physiological traits, senescence, tillage intensity

## Abstract

The aim of this study was to evaluate the effect of different intensities of tillage on the physiological characteristics and productivity of plants in crop rotation. Five tillage practices (DP—deep ploughing (22–24 cm); SP—shallow ploughing (16–18 cm); SH—shallow harrowing (8–10 cm); DH—deep harrowing (14–16 cm); and DD—direct drilling) were investigated in a long-term experiment in Dotnuva. The crop rotation was as follows: winter oilseed rape → spring wheat → spring barley → field pea → winter wheat. The simplification of conventional tillage negatively affected the photosynthetic indices of the majority of the crop rotation plants. The most favorable conditions for the photosynthetic processes in the plants were identified in the deep-ploughing treatment. The photochemical activity was negatively influenced and leaf senescence was accelerated under direct drilling. Direct drilling significantly decreased the grain yield of winter oilseed rape, spring wheat, and spring barley by 10.5%, 12.8%, and 17.2%, respectively, compared to deep ploughing. The grain yield of winter wheat was similar under deep ploughing and direct drilling; conversely, under shallow ploughing, shallow harrowing, and deep harrowing, the yield tended to decrease compared to deep ploughing.

## 1. Introduction

The tillage system influences the physical, chemical, and biological properties of soil [1,2,3]. Simplified tillage is used as a method for soil conservation and reducing labor costs [1,4]. Tillage impacts soil physical properties, such as pore-size distribution and total porosity [3]; soil structure [2]; and soil carbon sequestration capacity [5,6]. These soil characteristics are very important for providing favorable nutritional conditions for plants [5,7]. The tillage type can have both negative and positive effects on soil physical properties, as well as on plant productivity [4,8,9].

Tillage promotes root development [10]. The extent of the root zone and the distribution of root density govern the uptake of nutrients and water by plants, leading to increased crop yields [11]. The changes in nutritional conditions due to tillage intensity and soil compaction affect plant photosynthetic intensity [12]. Soil compaction has an adverse effect on plant physiological indices, such as chlorophyll content and chlorophyll fluorescence [13]. Tillage affects soil moisture and nutrient status, which in turn determine the plant senescence process [7,14,15]. This process finally leads to the death of vegetative and generative organs [16,17]. The most pronounced aspects of leaf senescence are the loss of chlorophyll pigments and the destruction of the photosynthetic apparatus [18], which reduce the efficiency of photosynthesis [16,17,19]. The processes related to the leaf senescence of cereals are important because they occur during grain filling, and, as was found in previous studies, premature senescence usually has a negative effect on yield [20,21]. There is still a lack of knowledge about the impact of tillage intensity on the physiological properties of plants grown in field conditions. The evaluation of the photosynthetic activity of plants under different tillage types can help explain the causality behind changes in plant productivity.

However, the impact of tillage intensity on crop physiological traits under field conditions in the Boreal region has not yet been investigated and understood. We hypothesized that by evaluating the photosynthetic activity of foliage and the differences in its parameters depending on the tillage method applied under field conditions, it would be possible to determine the most favorable conditions for photosynthetic processes in terms of tillage intensity. Our aim was to study the impact of different intensities of tillage on plant physiological traits under crop rotation.

## 2. Results

### 2.1. The Impact of Growth Stage and Tillage on Physiological Indices of Plants in Cropping System

A two-way ANOVA showed that during the generative development stages, the physiological indices were influenced by growth stage (factor A) (*p* ≤ 0.01) and tillage system (*p* ≤ 0.05, *p* ≤ 0.01) (Table 1). Growth stage (GS) was the main factor governing the total variability in the physiological indices data (the growth stages during the measurement of SPAD and Fv/Fm in different crops are provided in the Material and Methods section). GS determined 45.1%, 63.0%, 43.1%, and 83.4% of the total variability in SPAD in winter oilseed rape, spring wheat, spring barley, and field pea, respectively. Meanwhile, in winter wheat, GS explained only 14.7% of the SPAD differences between treatments. 

GS (factor A) was responsible for 74.9%, 70.9%, 45.7%, and 63.6% of the total variance in Fv/Fm in winter oilseed rape, spring wheat, field pea, and winter wheat, respectively. In spring barley, the influence of GS was lower (14.7%) but still statistically significant at *p* ≤ 0.01.

**Figure 1:**
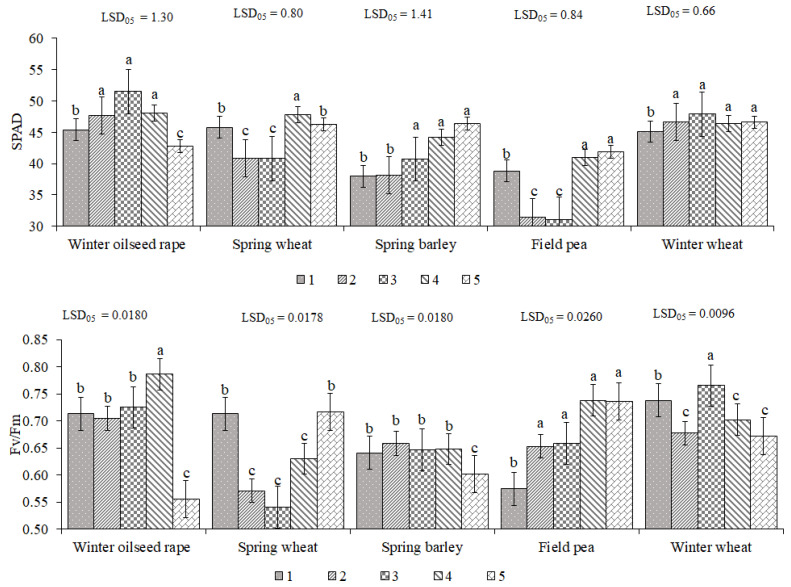
Growth stage (GS) effect on SPAD and Fv/Fm in crop-rotation plants averaged across tillage types. 1, 2, 3, 4, and 5 refer to measurements at different growth stages (as indicated in Table 5). The error bars show SE (standard error). Different letters denote statistically significant differences (at *p* ≤ 0.05 according to LSD) among treatments.

The dynamics of the SPAD and Fv/Fm values measured at the five different growth stages, averaged across tillage types, are provided in Figure 1. The results showed that compared to the values for the first measurement, SPAD coherently and significantly increased by 5.1–13.4% in winter oilseed rape until the fourth measurement and by 7.1–22.1% in spring barley and 2.9–6.2% in winter wheat throughout most of the measurements. The changes in SPAD in spring wheat and field pea were different. Compared to the first measurement, SPAD significantly decreased by 10.7–10.9% in spring wheat and by 19.1–19.8% in field pea at the second and third measurements. This decrease in SPAD may occurred due to the unfavorable humidity and temperature regime. However, during the last two measurements, SPAD significantly increased by 1.1–4.4% in spring wheat and by 5.7–8.0% in field pea.

Fv/Fm values varied according to the GS (Figure 1). In field pea, the Fv/Fm was low at the first measurement; however, it significantly increased by 13.8–28.6% in all following measurements. Except for spring wheat and field pea, the Fv/Fm values significantly decreased for the other crops in the final measurement.

It was found that the influence of tillage intensity (factor B) on the physiological indices was not very strong and significant in only a few tested cases (Table 1). Tillage type determined 5.3 and 5.5% of the total variability in SPAD in winter oilseed rape and spring wheat, respectively. Tillage had the highest and most significant impact on the SPAD of winter wheat and was responsible for 10.4% of the data variation. No significant effect of tillage intensity was found on SPAD in spring barley and field pea, or on Fv/Fm in all tested crops.

The effect of interactions between factors A × B on SPAD and Fv/Fm was insignificant in all tested cases.

**Figure 2:**
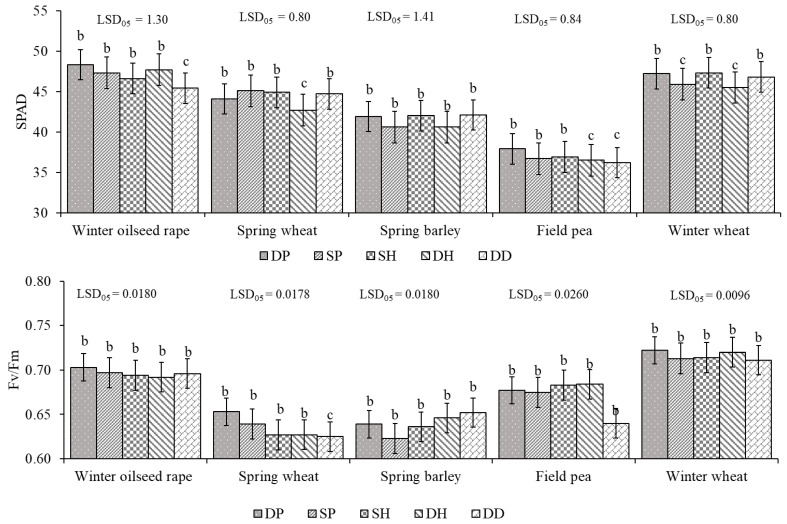
Tillage intensity effect on SPAD and Fv/Fm in crop-rotation plants averaged across GSs. DP—deep ploughing; SP—shallow ploughing; SH—shallow harrowing; DH—deep harrowing; and DD—direct drilling. The error bars show SE. Different letters denote statistically significant differences (at *p* ≤ 0.05 according to LSD) among treatments.

In terms of SPAD, winter crops reacted to tillage intensity more strongly than spring crops (Figure 2). In comparison with deep ploughing (DP), the simplification of tillage caused a decrease in SPAD for winter oilseed rape and winter wheat in most of the tested cases. Under the direct-drilling (DD) treatment, compared with DP, SPAD significantly decreased by 6.0% in winter oilseed rape. Meanwhile, in winter wheat, DD tillage decreased SPAD by 0.8% compared to DP. Shallow ploughing (SP) and deep harrowing (DH) had the highest negative impact on SPAD in winter wheat, with decreases of 2.8 and 3.6%, respectively.

Field pea was the most sensitive of the spring crops to the simplification of the tillage. All simplified-tillage treatments had a negative effect on SPAD for field pea. For DD, SPAD significantly decreased by 4.5% in comparison to DP. The application of DH also significantly decreased SPAD, by 3.7% compared to DP. SP and shallow harrowing (SH) decreased SPAD by 3.2 and 2.6%, respectively, compared to DP.

In spring wheat, SPAD significant decreased by 3.2% under DH in comparison with DP.

The influence of simplified tillage on Fv/Fm was negligible, with one exception: in spring wheat, Fv/Fm significant decreased by 4.3% under DD compared with DP (Figure 2).

### 2.2. Tillage Intensity Effect on Leaf Senescence in the Final Growth Stages of Crops

**Figure 3:**
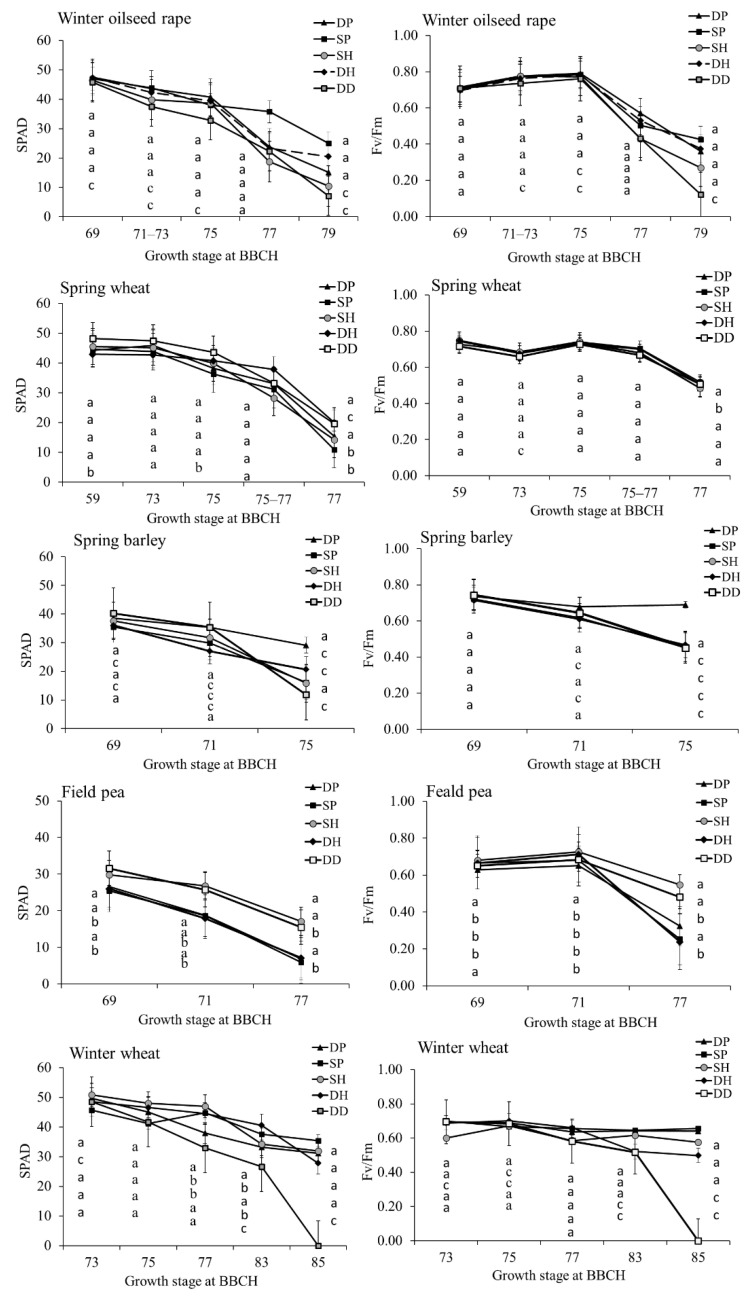
Tillage intensity effect on dynamics of SPAD and Fv/Fm in the final growth stages of the crops. DP—deep ploughing; SP—shallow ploughing; SH—shallow harrowing; DH—deep harrowing; and DD—direct drilling. Different letters denote statistically significant differences (at *p* ≤ 0.05 according to LSD) among treatments in the same order as in the legend. The error bars show SE.

In this study, leaf senescence was evaluated through the dynamics of the SPAD and Fv/Fm indices in the final GSs of the tested crops. We found that in the final GSs, i.e., after the flowering stage (BBCH 69), the photosynthetic indices decreased in all of the tested crops (Figure 3). In comparison with DP, the simplification of the tillage accelerated leaf senescence in some cases. Under DD, SPAD and Fv/Fm significantly decreased in winter oilseed rape in most cases, whereas in winter wheat, a significant decrease in the SPAD and Fv/Fm values was found in the last two measurements. In spring barley, compared with DP, all simplified-tillage treatments had a significant negative effect on the physiological indices for most of the measurements. 

The response of the spring crops to tillage intensity differed to that of the winter crops. In spring wheat and field pea, DD increased SPAD for most of the measurements in the final growth stages compared with DP (Figure 3). After the application of SH for field pea, SPAD also significantly increased in comparison with DP. The simplification of the tillage significantly increased the Fv/Fm values for field pea in most of the tested cases.

**Figure 4:**
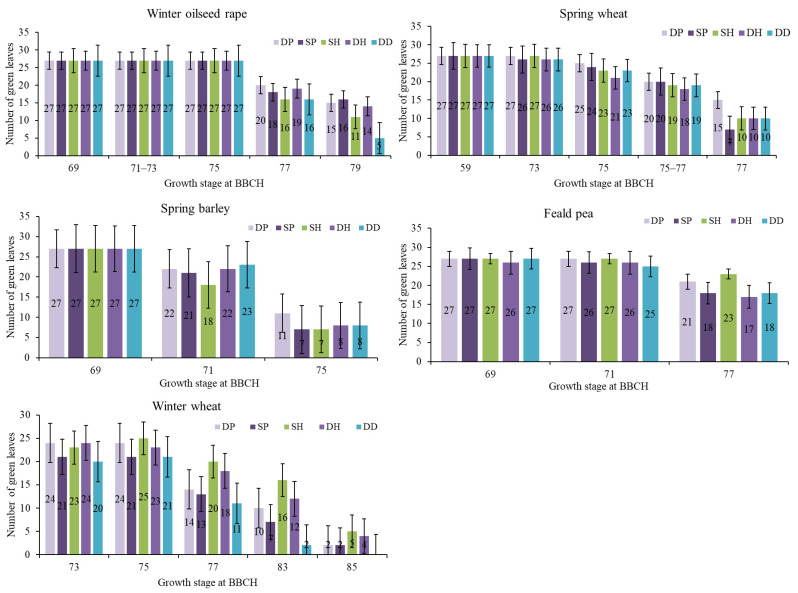
The number of green leaves in the final growth stages of the crops (the data presented indicate the sum of three plants, considering the 1st, 2nd, and 3rd leaves from the top). DP—deep ploughing; SP—shallow ploughing; SH—shallow harrowing; DH—deep harrowing; and DD—direct drilling. The error bars show SE.

The evaluation of the green-leaf number and loss dynamics in the final growth stages of the crops showed that the spring crops’ response to tillage simplification was less pronounced than that of the winter crops (Figure 4). According to the last measurement, at BBCH 75–77, SP had the most pronounced negative influence on leaf wilting and yellowing in spring wheat and spring barley. The highest leaf number for spring crops was found under DP.

In the winter crops, DD had the most pronounced negative influence on leaf senescence compared with DP. The application of SH in winter oilseed rape also decreased the number of leaves at BBCH 77 and BBCH 79. In winter wheat under SP, we also recorded noticeable leaf senescence from BBCH 73 until BBCH 85 compared with DP.

## 3. Discussion

### 3.1. The Influence of Tillage on Crop Physiological Parameters

Increasing the efficiency of the photosynthesis process and its adaptation to changeable environmental conditions is an important agronomic objective [22,23]. Understanding the photosynthesis process, its limiting factors, and the potential strategies for their circumvention is a promising approach for boosting the photosynthetic efficiency of crops [23,24]. Tillage affects the soil physical properties and temperature and water regimes, which are very important for providing favorable nutritional conditions for plants and for the photosynthesis process [2,3]. The deep tillage of soil may increase root penetration, stimulate root development, enhance nutrient accumulation, and improve crop yield [9,10]. The simplification of tillage can suppress the photosynthesis process [25]. The simplification of tillage limits the growth of the root system and, hence, the development and productivity of crops [12,13,14]. Soil compaction negatively influences leaf chlorophyll content [13]. Our findings also confirmed that a reduction in tillage intensity, compared with DP, caused a decrease in SPAD for most of the tested crops. The findings of the present study had a number of similarities with the data provided by other researchers [21,26,27], who evidenced the advantages of conventional tillage compared with direct drilling in terms of SPAD values. We found significant decreases in SPAD for winter oilseed rape and field pea under DD, whereas under DH tillage, significant differences were found for spring wheat, field pea, and winter wheat compared with DP. These results are contrary to the study of Hofmeijer et al. [4], wherein the SPAD of wheat under reduced tillage was significantly higher than under conventional tillage. In contrast, Liu and Wiatrak [28] proposed that tillage systems have no significant effect on SPAD.

The maximum quantum efficiency of PSII photochemistry (Fv/Fm) is mostly used to identify the efficiency of the photosynthetic apparatus [29]. It is an effective tool for discovering alterations in the function of the photosynthetic apparatus, which can be caused by environmental stress [24], changes in nutrient provision [30], and soil compaction [11,12]. Little is known about the impact of soil tillage methods on Fv/Fm in crops. Some studies have shown that the application of ploughing encouraged the photosynthesis process by increasing Fv/Fm in maize [31] and winter wheat [25], compared to subsurface tillage. Other studies suggest that Fv/Fm did not differ significantly between tillage systems [27]. We also found that simplified tillage did not have a significant effect on Fv/Fm in most crops compared with DP, except for significantly decreasing Fv/Fm in spring wheat under DD. The use of all other simplified-tillage systems decreased the Fv/Fm values for spring wheat compared with DP, whereas SP treatment decreased Fv/Fm in spring barley and, along with DD, in field pea.

### 3.2. The Influence of Tillage on Crop Leaf Senescence

Leaf senescence is an integrated response to age, developmental status, and environmental conditions [18]. Leaf senescence is an extremely regulated process during which nutritive substances are moved from the senescent leaf to other parts of the plant, leading to leaf death [7,19,32]. Under optimal growth conditions, senescence sets in following developmentally regulated processes, whereas, under unfavorable conditions, senescence can begin prematurely, engaging life-saving mechanisms such as early flowering and the rapid remobilization of nutrients from the senescent leaves to the seeds [33]. The beginning of senescence is essential for the transition to grain filling, therefore impacting grain yield [34]. The life duration of leaves, or the duration of photosynthetic activity, affects the amount of assimilates available for grain filling, thus influencing crop yield [19]. At the beginning of senescence, the leaves yellow due to chlorophyll degradation, and photosynthesis is slowed down [20]. Under unfavorable growth conditions, senescence processes can start earlier than normal [17]. Our experimental findings were in line with previous results [25], indicating that the simplification of tillage can diminish the photosynthesis process and lead to early leaf senescence. We found that tillage intensity had an influence on leaf senescence in all five tested crops; however, the influence differed between spring crops and winter crops. Our results agreed with those of previous studies [35,36] in that the lower the compaction of the soil, the longer the duration of plant vitality. Our results suggested that the simplification of traditional tillage, i.e., DP, had a variable influence on the photosynthetic indices of different plants. Spring crops responded to tillage simplification more strongly than winter crops: in the final growth stage (BBCH 77), spring wheat and field pea retained leaf greenness and vitality for longer under DD than DP. In winter oilseed rape and winter wheat, DD promoted the process of leaf senescence and leaf loss compared with DP. A longer period of functional photosynthesis with suspended leaf senescence could determine a higher accumulation of assimilates for grain filling and ultimately lead to increased crop yield [19,21].

### 3.3. The Influence of Tillage on Productivity and Quality of Crop-Rotation Plants

The effects of tillage on crop yield has been widely investigated [1,3,5,8,37]. Some studies have found a positive effect of no-tillage on grain yield, yield components, and quality parameters [5,15,38]. Hofmeijer et al. [4] reported that reduced tillage is a feasible alternative to ploughed systems; their results showed similar wheat grain yields under both tillage systems. Under minimum tillage, a high crop residue return can increase crop productivity due to the enhancement in soil structure and fertility [1]. However, our experimental findings were consistent with those of Macak et al. [6], who suggested that tillage simplification had a negative effect on crop productivity. The results of our study showed that under the application of DD, the grain yield significantly decreased for winter oilseed rape, spring wheat, and spring barley by 10.5%, 12.8%, and 17.2%, respectively, in comparison with DP (Table 2). The grain yield of field pea decreased under DD compared with DP, but the difference was insignificant. The productivity of winter wheat was similar under DP and DD, whereas SP, SH, and DH decreased its yield by 4.4% compared with DP.

We found that tillage simplification (SH and DH) significantly enhanced the oil content of winter oilseed rape, but that SH, DH, and DD had a negative and significant effect on the protein content of winter wheat. DH and DD significantly decreased the protein content in both spring wheat and spring barley compared with DP. Our findings were in line with those of Ali et al. [5] and Kulig et al. [26].

### 3.4. Relationship between Grain Yield, Grain Quality, Soil Temperature, and Plant Physiological Traits under Different Tillage Intensities

Tillage is an important factor affecting soil moisture, soil temperature, and nutrients [6,7,21,28,39], leading to alterations in plant growth and development [1,4,5,6], as well as plant physiological activity, including chlorophyll content, photosynthesis rate, and leaf area [25], which ultimately influence grain yield. The SPAD value has a strong relationship with grain yield, crop productivity, and production quality [16]. 

There is still lack of knowledge on the effect of tillage on plant physiological indices and their relationship with productivity. Some studies indicate that soil compaction under minimal tillage negatively influences the physiological traits of maize, including chlorophyll content [13]. Kulig et al. [26] found that conventional tillage promoted an increase in SPAD in spring wheat compared to simplified tillage.

We ascertained a correlation between physiological traits, crop yield, quality indices, and soil temperature averaged across crop rotations under different tillage intensities (Table 3). The data showed that SPAD was significantly (*p* ≤ 0.01) and positively correlated with grain yield, and the strength of this relationship was similar under all tillage intensity levels. On the contrary, other researchers found that the correlation between SPAD and wheat grain yield was not significant [26] or was absent in maize [28].

We found that SPAD was significantly (*p* ≤ 0.01) and positively correlated with protein content under DP, SP, and DH tillage. The correlation between SPAD and TGW was negative and significant (*p* ≤ 0.01) in all cases.

Fv/Fm was not correlated with crop yield, protein content, or TGW; however, a significant (*p* ≤ 0.01) relationship was found with HLM under all tillage regimes except DD.

Increasing air temperature and soil temperature and changes in precipitation patterns as a consequence of climate change affect crop production in agricultural ecosystems [40]. Muñoz-Romero et al. [39] found that the soil temperature was higher under conventional tillage than no tillage. Changes in the amount and frequency of precipitation may influence the availability of nutrients for crops, resulting in leaf senescence [41]. In our study, SPAD was negatively correlated (*p* ≤ 0.01) with soil temperature in most of the tested cases. Under all tillage intensity levels, no relationship was found between Fv/Fm and soil temperature. 

## 4. Materials and Methods

### 4.1. Site and Soil Description

A long-term field experiment was carried out at the Institute of Agriculture, Lithuanian Research Centre for Agriculture and Forestry in Central Lithuania (55°23′50″ N and 23°51′40″ E) over five growing seasons, 2012–2016. The soil of the experimental site was classified as an *Endocalcari-Epihypogleyic* Cambisol. The loam soil was close to neutral (pH_KCl_ 6.3–6.8, measured potentiometrically); medium rich in humus (2.2–2.7%, Tyurin method); rich in available phosphorus (160–250 mg kg^−1^, A-L method); and rich in available potassium (180–300 mg kg^−1^, A-L method).

### 4.2. Experimental Details and Agronomic Management

The field experiment was arranged in four blocks (replications) with five tillage treatments of different intensities: DP—deep ploughing (22–24 cm); SP—shallow ploughing (16–18 cm); SH—shallow harrowing (8–10 cm); DH—deep harrowing (14–16 cm); and DD—direct drilling. Each tillage plot was 10 m wide and 21 m long. The crop rotation was as follows: winter oilseed rape—spring wheat—spring barley—field pea—winter wheat. The crop husbandry details are provided in Table 4.

### 4.3. Measurements of Physiological Parameters

Leaf chlorophyll index (SPAD) was measured non-destructively with a portable Minolta SPAD 502 Chlorophyll Meter (Minolta Camera Co. Ltd., Osaka, Japan). The measurements were made in the middle part of fully expanded, randomly selected leaves of 40 plants per treatment (10 plants per plot x 4 blocks). SPAD measurements were carried out from 10 am until 2 pm (local time) on clear days five times for all crop-rotation plants (Table 5). Growth stages according to the BBCH scale were identified following Meier [42].

To obtain the maximum quantum efficiency of PSII photochemistry (Fv/Fm), a multi-functional pulse-modulated handheld chlorophyll fluorometer (model OS-30p; Opti-Sciences, Inc., Hudson, NH, USA) was used to measure chlorophyll-α fluorescence in vivo. Fv/Fm was read directly on the chlorophyll fluorometer after a short period of adaptation to the dark [43]. Leaves of plants were allowed to adapt to the darkness for 1 min using light-withholding clips. Fv/ Fm measurements were made on the 1st fully expanded and randomly selected leaves of 5 plants per plot × 3 blocks (15 plants per treatment), five times per growing season (Table 5). 

### 4.4. Measurement of Crop Leaf Senescence

A total of 3 plants in each plot were randomly selected and marked with a bright strip. The same leaves were assessed at each measurement timepoint until they withered. The green-leaf number and loss dynamics were evaluated. SPAD and Fv/Fm were measured in the 1st, 2nd, and 3rd leaves from the top. The data were collected during the final growth stage of the crops (Table 6).

### 4.5. Measurement of Soil Temperature

The soil temperature was measured using a digital long-stem thermometer (Spectrum Technologies, Aurora, IL, USA). The soil temperature at the 5 cm soil layer was measured at the same growth stages (Table 5) and times as the physiological parameters.

### 4.6. Grain/Seed Yield (GY) and Grain/Seed Quality Analyses

The plots were harvested at complete maturity with a plot harvester (“Wintersteiger Delta”, Arnstadt, Germany). The harvested area totaled 36.9 m^2^. Grain/seed yield as t ha^−1^ was adjusted to 14% moisture content for cereals and field pea and to 9% moisture content for winter oilseed rape.

The thousand grain weight (TGW) was counted with a Contador seed counter (“Pfeuffer”, Kitzingen, Germany) from four samples of 250 seeds per plot. Protein content and oil content from each plot were measured using an Infratec 1241 grain analyzer (FOSS, Hilleroed, Denmark).

### 4.7. Statistical Analysis

Two-way ANOVA was used to determine the effects of growth stage and tillage system on the physiological indices. Fisher’s test was also used. Statistical significance was evaluated at the *p* ≤ 0.05 and *p* ≤ 0.01 probability levels. Standard statistical procedures were used for calculating simple correlation coefficients among physiological traits, yield, quality indices, and soil temperature under different tillage systems. The statistical analysis was carried out using STAT ENG software for Excel version 1.55 from the statistical data-processing package SELEKCIJA.

### 4.8. Meteorological Conditions

**Figure 5:**
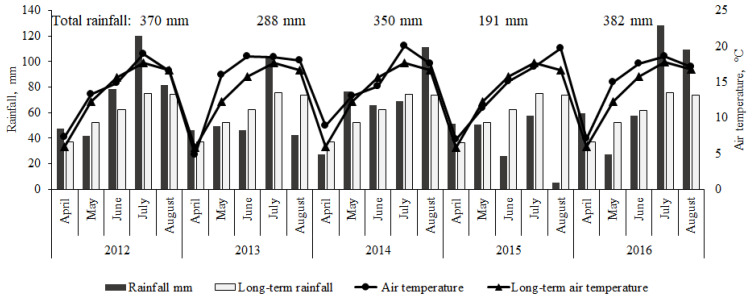
The distribution of rainfall and temperature during the growing seasons.

Rainfall and mean air temperature data over the five growing seasons (provided by the Dotnuva weather station, located about 500 m from the experimental field) are provided in Figure 5. The conditions of the plant growing seasons were described using the hydrothermal coefficient as the agrometeorological indicator, which was calculated according to the formula:

HTC = Σ p/0.1 Σ t,(1)

where Σ p represents the sum of precipitation (mm) during the test period, when the average daily air temperature was above 10 °C, and Σ t denotes the sum of active temperatures (°C) during the same period.

HTC > 1.6 indicates excessive irrigation; HTC = 1.0–1.5 optimal irrigation; HTC = 0.9–0.8 weak drought; HTC = 0.7–0.6 moderate drought (arid); HTC = 0.5–0.4 heavy drought; and HTC < 0.4 very heavy drought [44].

Rainfall differed between the growing seasons, and the amount of rainfall totaled 370 mm (HTC = 1.6), 288 mm (HTC = 1.2), 350 mm (HTC = 1.6), 191 mm (HTC = 1.0), and 382 mm (HTC = 1.3) in the 1st, 2nd, 3rd, 4th, and 5th seasons, respectively.

## 5. Conclusions

The results of the five-year study revealed that the simplification of traditional tillage had a varied influence on the photosynthetic indices of most of the crop-rotation plants. Direct drilling (DD) tended to reduce the Fv/Fm values in field pea, winter wheat, winter oilseed rape, and spring wheat leaves. Under shallow ploughing (SP), in most tested cases, Fv/Fm decreased in winter wheat, spring wheat, and winter oilseed rape. In terms of SPAD, winter crops responded more strongly to tillage simplification than spring crops. Compared to deep ploughing (DP), all simplified-tillage systems significantly reduced the SPAD values in winter wheat. In winter oilseed rape, direct drilling (DD) and shallow harrowing (SH) significantly reduced SPAD, while shallow plowing (SP) and deep harrowing (DH) decreased SPAD. Compared to DP, SP and DD significantly reduced SPAD in field pea. In spring wheat, DD had a significant positive effect on SPAD values. DD accelerated the senescence process in winter crops.

The differences in the photosynthetic activity indicators depending on the applied tillage types suggested that the most favorable conditions for photosynthetic processes in plants were provided by DP.

In comparison with DP, DD application significantly decreased the grain yield of winter oilseed rape, spring wheat, and spring barley by 10.5%, 12.8%, and 17.2%, respectively; meanwhile, the grain yield of field pea decreased under DD, but the difference was insignificant. The grain yield of winter wheat was similar under DP and DD, whereas SP, SH, and DH tended to decrease the yield compared with DP.

## Figures and Tables

**Table 1:** Contribution (% of sum of squares) of growth stage and tillage intensity and their interaction to total variance in physiological indices of plants in cropping system.

Crop	Indices	Growth Stage (A)	Tillage Intensity (B)	A × B	Total
Winter oilseed rape	SPAD	45.1 **	5.3 *	5.2	55.6
	Fv/Fm	74.9 **	0.2	5.5	80.6
Spring wheat	SPAD	63.0 **	5.5 **	3.6	72.1
	Fv/Fm	70.9 **	1.6	7.1	79.6
Spring barley	SPAD	43.1 **	1.8	13.0	57.8
	Fv/Fm	16.0 **	3.9	13.3	33.2
Field pea	SPAD	83.4 **	1.3	2.5	87.3
	Fv/Fm	45.7 **	3.2	12.1	31.0
Winter wheat	SPAD	14.7 **	10.4 *	12.1	37.1
	Fv/Fm	63.6 **	2.2	8.4	74.2

* and **—significant at *p* ≤ 0.05 and *p* ≤ 0.01, respectively, according to Fisher’s test.

**Table 2:** The influence of tillage on productivity and quality indices of crop-rotation plants. Values are presented as mean ± SE of four replicates.

Crop	Tillage	Grain Yield (t ha^−1^)	Protein (%)	TGW (g)	HLW (kg hl^−1^)
Winter oilseed rape	DP	3.72 ± 0.10 b	^§^ 45.1 ± 0.24 b	6.37 ± 0.10 b	-
	SP	3.54 ± 0.11 b	^§^ 45.2 ± 0.29 b	6.35 ± 0.08 b	-
	SH	3.26 ± 0.15 c	^§^ 45.7 ± 0.17 a	6.37 ± 0.07 b	-
	DH	3.57 ± 0.01 b	^§^ 45.9 ± 0.22 a	6.38 ± 0.05 b	-
	DD	3.33 ± 0.10 c	^§^ 45.6 ± 0.19 b	6.35 ± 0.02 b	-
	LSD_05_	0.294	0.60	0.230	
Spring wheat	DP	5.86 ± 0.11 b	13.1 ± 0.26 b	40.0 ± 0.31 b	75.4 ± 0.56 b
	SP	6.02 ± 0.17 b	13.1 ± 0.23 b	39.9 ± 0.60 b	75.8 ± 0.50 b
	SH	5.98 ± 0.18 b	13.2 ± 0.18 b	40.6 ± 0.59 b	75.3 ± 1.03 b
	DH	5.97 ± 0.11 b	12.5 ± 0.17 c	39.5 ± 0.34 b	74.5 ± 0.84 b
	DD	5.11 ± 0.22 c	13.2 ± 0.19 b	39.6 ± 0.30 b	73.8 ± 0.77 b
	LSD_05_	0.530	0.44	1.30	2.56
Spring barley	DP	6.91 ± 0.33 b	11.8 ± 0.18 b	43.9 ± 0.41 b	61.0 ± 0.51 b
	SP	6.72 ± 0.26 b	12.5 ± 0.27 a	44.0 ± 0.29 b	60.8 ± 0.50 b
	SH	7.01 ± 0.17 b	11.8 ± 0.13 b	42.9 ± 0.24 c	61.0 ± 0.17 b
	DH	6.51 ± 0.07 b	11.3 ± 0.2 b	41.8 ± 0.11 c	60.3 ± 0.42 b
	DD	5.72 ± 0.49 c	10.9 ± 0.14 c	43.6 ± 0.16 b	61.0 ± 0.45 b
	LSD_05_	0.981	0.63	0.72	1.44
Field pea	DP	2.29 ± 0.18 b	- *	223.7 ± 4.46 b	87.8 ± 0.56 b
	SP	2.57 ±0.18 b	-	222.9 ± 3.09 b	86.8 ± 0.52 b
	SH	2.07 ± 0.09 b	-	222.7 ± 3.55 b	87.2 ± 0.41 b
	DH	2.40 ± 0.17 b	-	227.5 ± 2.32 b	88.2 ± 0.29 b
	DD	2.00 ± 0.26 b	-	229.8 ± 5.81 b	87.0 ± 1.01 b
	LSD_05_	0.499		12.04	1.85
Winter wheat	DP	8.26 ± 0.15 b	12.8 ± 0.05 b	37.4 ± 0.54 b	81.2 ± 0.43 b
	SP	7.92 ± 0.13 b	12.7 ± 0.13 b	38.4 ± 0.92 b	80.5 ± 1.19 b
	SH	7.89 ± 0.04 b	12.5 ± 0.09 c	39.2 ± 0.86 b	80.3 ± 0.98 b
	DH	7.90 ± 0.27 b	12.4 ± 0.11 c	38.7 ± 1.13 b	80.2 ± 0.71 b
	DD	8.21 ± 0.16 b	12.3 ± 0.05 c	38.6 ± 0.63 b	81.7 ± 0.54 b
	LSD_05_	0.438	0.20	2.32	2.21

TGW—thousand grain weight; HLW—hectolitre weight; DP—deep ploughing; SP—shallow ploughing; SH—shallow harrowing; DH—deep harrowing; DD—direct drilling; ^§^ oil content; *—no data. Different letters in each column denote statistically significant differences (at *p* ≤ 0.05 according to LSD) among treatments.

**Table 3:** Correlation coefficients among physiological traits, yield, quality indices, and soil temperature averaged across crop rotations under different tillage intensities.

Tillage Intensity	Indices	Fv/Fm	GY	Protein ^†^	TGW	HLM ^§^	T_0–5 cm_
DP	SPAD	0.331 **	0.576 **	0.413 **	−0.550 **	−0.113	−0.297 **
	Fv/Fm		0.058	0.234 *	0.057	0.287 **	−0.242 *
	GY			−0.844 **	−0.912 **	−0.502 **	−0.538 **
	Protein ^†^				−0.99 **	0.739 **	−0.391 **
	TGW					0.645 **	0.470 **
	HLM						0.290 **
SP	SPAD	0.198	0.584 **	0.314 **	−0.572 **	−0.092	−0.213
	Fv/Fm		0.081	0.204	0.073	0.368 **	−0.210
	GY			−0.892 **	−0.929 **	−0.509 **	−0.540 **
	Protein ^†^				−0.989 **	0.285 *	−0.413 **
	TGW					0.626 **	0.484 **
	HLM						0.331 **
SH	SPAD	0.252 *	0.624 **	0.204	−0.624 **	−0.170	−0.338 **
	Fv/Fm		−0.060	0.204	0.161	0.290 **	−0.114
	GY			−0.921 **	−0.948 **	−0.596 **	−0.504 **
	Protein ^†^				−0.995 **	0.672 **	−0.391 **
	TGW					0.662 **	0.426 **
	HLM						0.316 **
DH	SPAD	0.205	0.554 **	0.440 **	−0.513 **	−0.155	−0.262 *
	Fv/Fm		0.029	0.164	0.139	0.297 **	−0.143
	GY			−0.878 **	−0.929 **	0.526 **	−0.546 **
	Protein ^†^				−0.996 **	0.854 **	−0.406 **
	TGW					0.690 **	0.500 **
	HLM						0.320 **
DD	SPAD	0.192	0.602 **	0.097	−0.623 **	−0.195	−0.270 *
	Fv/Fm		0.184	0.191	−0.110	0.042	−0.187
	GY			−0.720 **	−0.825 **	−0.277 **	−0.530 **
	Protein ^†^				−0.995 **	0.664 **	−0.423 **
	TGW					0.639 **	0.413 **
	HLM						0.148

^§^—without winter oilseed rape data; ^†^ protein in cereals, oil content in winter oilseed rape; T_0–5 cm_—temperature °C in soil 0–5 cm layer; SPAD—chlorophyll index; Fv/Fm—maximum quantum efficiency; GY—grain yield; TGW—thousand grain weight; HLM—hectolitre mass; DP—deep ploughing; SP—shallow ploughing; SH—shallow harrowing; DH—deep harrowing; DD—direct drilling; * and **—significant at *p* ≤ 0.05 and *p* ≤ 0.01, respectively.

**Table 4:** Details of crop husbandry adopted in the study.

Crops	Sowing Time	Cultivar	Seed Rate (Viable Seeds mln ha^−1^)	Crop Density (Plants m^2^)	Fertilizer NPK (kg ha^−1^)
Winter oilseed rape	24 August 2012	Komando	0.7	45	N_194_ P_53_ K_105_
Spring wheat	2 May 2013	Granary	5.0	256	N_152_ P_48_ K_102_
Spring barley	17 April 2014	Grace	4.0	294	N_132_ P_48_ K_102_
Field pea	14 April 2015	Pinocchio	1.0	69	N_14_ P_56_ K_119_
Winter wheat	10 September 2016	Ada	4.5	395	N_184_ P_56_ K_119_

**Table 5:** Growth stages at which measurements of SPAD and Fv/Fm were carried out in different crops.

	Measurement No.				
	1	2	3	4	5
Crop	Growth Stage (BBCH)				
Winter oilseed rape	60–61	63–65	69	71–73	75–78
Spring wheat	32	39–41	51–53	59	71
Spring barley	31	39	49	59	69
Field pea	15	17–18	39	61	69
Winter wheat	32	41–43	55	61	63

**Table 6:** Growth stages at which the measurements of senescence (SPAD and Fv/Fm) were carried out.

Crops	Growth Stage (BBCH)				
Winter oilseed rape	69	71–73	75	77	79
Spring wheat	59	73	75	75–77	77
Spring barley	69	71	75	-	-
Field pea	69	71	77	-	-
Winter wheat	73	75	77	83	85

## Data Availability

Not applicable.
